# Vitamin D Status and Gastric Cancer: A Cross-Sectional Study in Koreans

**DOI:** 10.3390/nu12072004

**Published:** 2020-07-06

**Authors:** Jung Hyun Kwak, Jean Kyung Paik

**Affiliations:** Department of Food and Nutrition, Eulji University, Seongnam 13135, Gyeonggi-do, Korea; hyun4615@hanmail.net

**Keywords:** vitamin D, gastric cancer, KNHANES, cross-sectional study, Korea

## Abstract

Sufficient vitamin D levels are associated with reduced *Helicobacter pylori* infections, which can cause gastric carcinogenesis. We examined associations between vitamin D concentrations and gastric cancer (GC) prevalence in a Korean population. We analyzed data of 33,119 adults using serum 25-hydroxyvitamin D (25(OH)D) concentrations as a biomarker of vitamin D status. Participants were classified with GC if previously diagnosed as such by a physician. After controlling for age, sex and body mass index (model A), odds ratio (OR) for GC was 0.81 (95% confidence interval [CI]: 0.70, 0.95), with a 5-ng/mL increment in total 25(OH)D concentrations. In fully adjusted models (model B), the OR for GC was 0.84 (95% CI: 0.72, 0.98), with a 5-ng/mL increment in total vitamin D. Following the classification of vitamin D concentrations into three categories or for GC in model A was 0.52 (95% CI: 0.30, 0.92) comparing between higher (≥20 ng/mL) and lower (<12 ng/mL) total 25(OH)D concentrations. In model B OR for GC was 0.57 (95% CI: 0.32, 1.00) comparing between higher and lower total 25(OH)D concentrations. Our results suggested that high vitamin D concentration was associated with lower ORs of GC in Korean adults.

## 1. Introduction

Worldwide, gastric cancer (GC) ranks fifth in cancer incidence [1], with 1.3 million (1.2–1.4 million) incident cases of GC in 2015. In Korea, GC ranks second in cancer incidence, and with a 35.8 per 100,000 incidence rate in 2014 [2].

Some risk factors for the development of GC, such as age and sex, are not modifiable, whereas others such as smoking and *Helicobacter pylori* infection are potentially modifiable [3]. In addition, in a study of factors affecting GC, Ren et al. reported that patients with sufficient vitamin D had a lower overall mortality than patients with vitamin D deficiency in GC patients [4]. An experimental study suggested that 1,25-(OH)D_3_ can induce apoptosis in GC cells, suggesting its use in cancer therapy [5]. A recent meta-analysis reported that sufficient vitamin D could be associated with a decrease in *H. pylori* infection, a major risk factor for GC [6]. A review study by Du et al. suggested that vitamin D may inhibit viability, proliferation and metastasis of gastric cancer cells and inhibit *Helicobacter pylori* infection and *Helicobacter*-related gastric cancer [7]. Conversely, another meta-analysis study reported no statistically significant relationship between serum vitamin D concentrations and risk of GC, for the whole population nor for gender-stratified analyses [8]. The findings used in this meta-analysis reviewed a small number of studies, and the results were inconsistent. Although vitamin D deficiency is high in Koreans [9], few studies have been conducted on GC. Eom et al. performed a case–control study, they did not find an inverse association between vitamin D status and risk of GC [10]. However, these associations require further research as no study was based on data from the Korea National Health and Nutrition Examination Survey (KNHANES). Therefore, this study aimed to investigate the associations between vitamin D status and the prevalence of GC in a Korean population.

## 2. Materials and Methods

### 2.1. Study Population

Data on participants aged ≥20 years were collected from the KNHANES from 2008–2014. The KNHANES, conducted by the Centers for Disease Control and Prevention (CDC), is a health-related survey to assess the health and nutritional status of the South Korean population. The survey uses a complex, multi-staged, stratified, clustered design and included household performed interviews including demographic, socioeconomic and dietary questionnaires, followed by a medical interview and physical examination with medical, dental and physiological measurements [11].

**Figure 1:**
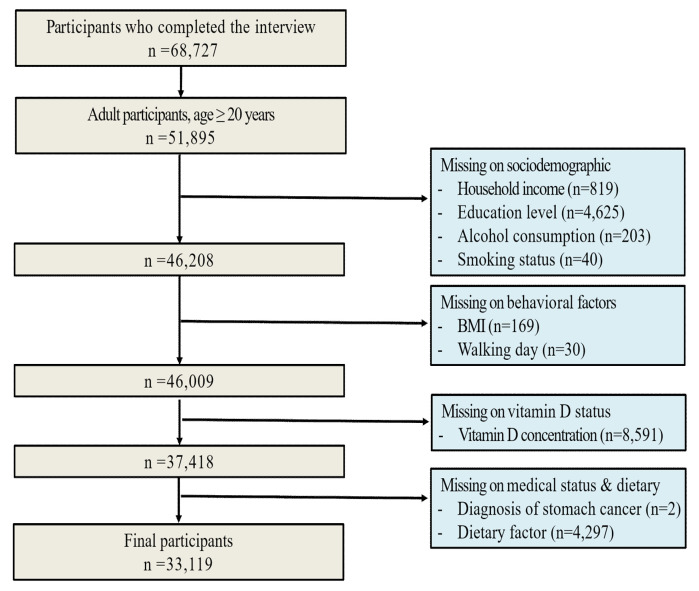
Description of the study population.

Among the initial enrollment of 68,727 subjects in the KNHANES form 2008–2014, we enrolled 51,895 adults aged ≥20. We excluded subjects with missing information on household income (n = 819), education level (n = 4625), alcohol consumption (n = 203), smoking status (n = 40), BMI (n=169), frequency of walking (per week) (n = 30), vitamin D concentrations (n = 8591) and other covariates (n = 4299). Finally, 33,119 subjects were included in our analysis. Figure 1 provides a description of the study participants. The study protocol was approved by the National Centers for Health Statistics Research Ethics Review board (2008-04EXP-01-C, 2009-01CON-03-2C, 2010-02CON-21-C, 2011-02CON-06-C, 2012-01EXP-01-2C, 2013-07CON-03-4C, 2014-12EXP-03-5C). Written informed consent was obtained from all KNHANES participants. NHANES is a publicly available data set. Additional details on study procedures, data documentation and questionnaires have been published previously [11].

### 2.2. Vitamin D Concentrations

Blood samples were collected following an 8-h fast. Blood samples were immediately refrigerated and then shipped to the central testing institute (NeoDin Medical Institute, Seoul, South Korea) for analysis. Serum 25(OH)D concentrations were measured using a 1470 Wizard gamma counter (PerkinElmer, Turku, Finland) and radioimmunoassay (RIA) (DiaSorin, Stillwater, MN, USA). For statistical analysis, 25(OH)D concentrations were categorized into three groups, namely <12-ng/mL (deficient), 12–19.9-ng/mL (suboptimal) and ≥20-ng/mL (sufficient), based on cut-points by the Institute of Medicine (US) [12].

### 2.3. Gastric Cancer

Participants were classified as having GC if they reported that they were diagnosed by a physician. The participants were also classified according to whether they currently had GC.

### 2.4. Covariates

We considered participant age, sex, body mass index (BMI), household income, education level, alcohol consumption, smoking status, frequency of walking (per week) and dietary intake (total energy, calcium, vitamin A) as potential confounders. Education was classified as less than elementary school (reference), middle school, high school and more than college. Smoking status was classified as never (reference), former or current smoker. Alcohol consumption was categorized as never (reference), ≤ once per month, 2–4 times/month or ≥2 times/week. Information on dietary intakes (total energy, calcium, vitamin A) was obtained via a 24-h dietary recall interview. Dietary food records were collected for one day. BMI was classified as underweight; <18 kg/m^2^ (reference), normal weight; 18–22.9 kg/m^2^, overweight; 23–24.9 kg/m^2^ or obesity; ≥25 kg/m^2^. Frequency of walking was categorized as never (reference), 1–2 times/week, 3–4 times/week, 5–6 times/week or every day.

### 2.5. Statistical Analysis

Data analyses were performed using SPSS version 20.0 (IBM corp., New York, NY, USA) to account for the complex survey design and sample weights of KNHANES 2008–2014. *P*-values were two-sided; values <0.05 were considered statistically significant.

In descriptive analysis, we presented continuous variables as weighted means and standard error (SE) and categorical variables as weighted percentages (%). We also performed a survey *t*-test for continuous variables and survey (Rao–Scott) χ^2^ test for categorical variables.

The associations between vitamin D concentrations and GC were examined using multiple logistic regression models. We evaluated two sequential models: model A adjusted for age, sex and BMI and model B adjusted for model A covariates plus education, household income, smoking status, alcohol consumption and dietary factor (intake of total energy, calcium and vitamin A).

## 3. Results

### 3.1. Descriptive Statistics

Overall, 33,119 participants were available for analysis. Table 1 shows the general characteristics stratified based on the diagnosis of GC. Among participants with a diagnosis of GC, the mean (± SE) age was 61.8 (±1.25) years. Males accounted for 61.3% of the study participants and the mean total vitamin D concentration was 17.4 (±0.59) ng/mL. Among participants without a diagnosis of GC, the mean (± SE) age was 44.5 (±0.17) years. Males accounted for 51.0% of the study population, and the mean total vitamin D concentration was 17.5 (±0.10) ng/mL. Compared with participants without a diagnosis of GC, those with a diagnosis of GC were significantly older and had significantly lower diastolic blood pressure (DBP), BMI and total energy intake. Moreover, household income, alcohol consumption, smoking status and education level significantly differed depending on the diagnosis of GC.

Table 2 is a summary of participant characteristics according to vitamin D status. Compared with participants who were deficient, those with sufficient total 25(OH)D concentrations were likely to be older, had higher male distribution, systolic blood pressure (SBP), DBP and consumed more total energy and calcium. Furthermore, household income, alcohol consumption, smoking status, education level, BMI category and frequency of walking significantly differed depending on the vitamin D status.

### 3.2. Vitamin D and GC

Table 3 shows the results of the associations between vitamin D and GC. After controlling for age, sex and BMI (model A), the odds ratio (OR) for GC was 0.81 (95% confidence interval [CI]: 0.70, 0.95) with a 5-ng/mL increment in total vitamin D concentrations. After adjusting for model A covariates plus education, household income, smoking status, alcohol consumption and dietary factor (intake of total energy, calcium, vitamin A) (model B), the OR for GC was 0.84 (95% CI: 0.72, 0.98) with a 5-ng/mL increment in total vitamin D concentrations. Additionally, vitamin D concentrations were classified into three categories and analyzed. In model A, higher total vitamin D concentrations were associated with a gradually lower OR for GC (*p* for trends = 0.030), and the OR for GC was 0.52 (95% CI: 0.30, 0.92) in the higher total vitamin D concentrations (≥20 ng/mL) compared with that in the lower total vitamin D concentrations (<12 ng/mL). In the fully adjusted models (model B), the OR for GC was 0.57 (95% CI: 0.32, 1.00) in the higher total vitamin D concentrations (≥20 ng/mL) compared with that in the lower total vitamin D concentrations (<12 ng/mL).

## 4. Discussion

This study evaluated the associations between vitamin D status and the prevalence of GC using the KNHANES. In this nationally representative cross-sectional survey of adults, our findings showed that vitamin D sufficiency was inversely associated with a prevalence of GC. Vitamin D deficiency has emerged as a global public health problem [13] and causes several health problems such as osteomalacia, osteoporosis, myopathy, autoimmune disease, hypertension, diabetes and cancer [14]. Experimental studies have identified the effects of vitamin D on cancer [5,15,16]. Deeb et al. reported that the antitumor effects of vitamin D involve mechanisms that are associated with G0/G1 arrest, differentiation, induction of apoptosis and inhibition of tumor angiogenesis [14]. Furthermore, Feldman et al. suggested that vitamin D_3_ regulates multiple signaling pathways involved in proliferation, apoptosis, differentiation, inflammation, invasion, angiogenesis, metastasis and microRNA expression, and it may affect cancer development, growth and cancer stem cell biology [15]. Pen et al. investigated whether vitamin D had any effects on GC cells and found that bioactive vitamin D significantly promoted apoptosis in the undifferentiated GC cell line HGC-27 and could induce PTEN expression (tumor suppressor gene) via VDR [5].

Several studies in humans have hypothesized that serum vitamin D concentrations may affect the risk of cancer. However, the findings are inconsistent [8,10,17]. A systematic review and meta-analysis, which analyzed 44,165 cases from 64 studies, suggested that a higher vitamin D status had a lower mortality rate (26%) and a lower disease progression rate (16%) in cancer patients [17]. Conversely, a meta-analysis study, which analyzed 3022 cases from seven studies, reported no statistically significant relationship between serum vitamin D concentrations and risk of GC [8]. A recent case–control study suggested no association between vitamin D intake and the risk of GC in Koreans [10]. However, there was no statistical significance in their finding, the risk of GC was lower at 6%–7% in the middle or highest vitamin D intake groups compared with the lowest group. Moreover, a retrospective case–control study reported that a positive relationship between vitamin D deficiency and gastric adenocarcinoma [18]. However, there are limited data regarding GC in Korea.

To our knowledge, this is the first study to examine the association between vitamin D status and the prevalence of GC in Korea. However, the prevalence rate of GC has been steadily decreasing worldwide [19], GC ranks second in cancer incidence and the third most common cause of cancer-related mortalities in Korea [20]. Chronic *H. pylori* infection was the major risk factor for GC [21]. A recent meta-analysis reported that sufficient vitamin D could be associated with decreased in *Helicobacter pylori* infection [6], and they found that vitamin D concentrations in *H. pylori*-positive patients were lower than those in *H. pylori*-negative patients. Furthermore, subjects with vitamin D deficiency was associated with lower success rates of *H. pylori* eradication [22,23]. According to a mechanistic study of vitamin D on *H. pylori*, vitamin D may have antibacterial activity [24,25] and immune response [26]. However, mechanism studies involving vitamin D and GC require further validation.

Results of previous studies support our results shown in the fully adjusted models. The OR for GC was 0.84 (95% CI: 0.72, 0.98) with a 5-ng/mL increment in total vitamin D concentration. In addition, the OR for GC was 0.57 (95% CI: 0.32, 1.00) in the higher total 25(OH)D concentrations (≥20 ng/mL) compared with that in the lower total 25(OH)D concentrations (<12 ng/mL). We further analyzed the association between gastric cancer and gastric cancer-related risk factors in the subjects excluded for missing data on vitamin D levels (n = 8591). The final number for the analysis was 7687, excluding those without data on diet and gastric cancer diagnosis. In this subgroup, we adjusted for sex, age, BMI, alcohol consumption, smoking status, education level, household income, frequency of walking and dietary factors. Smoking and alcohol intake did not show a significant difference from the ORs of GC; the ORs of GC was 1.73 (1.42, 2.11) when age was increased in 10-year increments. When BMI was increased by five units, the ORs of GC was 0.334 (0.23, 0.48) (data not shown). Since vitamin D levels were high in obese people across our entire dataset, it is likely that people with high vitamin D levels were in subgroups that were missing data on the vitamin D levels. In addition, we analyzed the association between current prevalence of gastric cancer and vitamin D level at the time of the investigation (Supplemental Table S1). However, there were too few subjects with current gastric cancer for a robust analysis. Thus, case–control or cohort studies are needed in the future for more accurate results.

The major strengths of this study include the use of data from a representative sample of Korea from 2008 to 2014, which supports the increased power of our findings. Additionally, we adjusted our models for several potential confounding factors, including sociodemographic, behavioral and dietary factors, and our results persisted after adjustment for those factors. In particular, we reviewed the literature and adjusted for calcium [27] and vitamin A intakes [28] that could affect the prevalence of GC. Nevertheless, this study had some limitations. First, because of the study’s cross-sectional design, we cannot demonstrate causality. Second, although we controlled for several potential confounders, there is a possibility of residual confounding factors. Since there were no data available in KNHANES 2008–2014, we did not control for the timing of the vitamin D level measurement as a covariate. In addition, we did not control for supplement use. As questions regarding supplements frequently changed, we could not integrate them. For example, in 2008, the questionnaire asked specifically if they had taken any nutritional supplements, such as vitamins and minerals; whereas, in 2010–2012, the questionnaire asked if they had taken dietary supplements more than once per week in the last month. Third, we also analyzed a Korean population. Thus, this result should be cautiously considered when generalized to other ethnic populations. Fourth, dietary data measured once were used owing to the limitations of KNHANES data.

## 5. Conclusions

We found that vitamin D sufficiency was associated with a lower prevalence of GC. However, further large-scale prospective studies are needed to clarify the epidemiologic relationship between vitamin D and the risk of GC.

## Figures and Tables

**Table 1:** General characteristics stratified by diagnosis of gastric cancer.

	Diagnosis of Gastric Cancer		
**Characteristic**	Yes	No	*p*
**Sex, No. (%) of participant**			
**Men**	138 (61.3)	13,383 (51.0)	0.057
**Women**	80 (38.7)	19,518 (49.0)	
**Age (years)**	61.8 (±1.25)	44.5 (±0.17)	<0.001
**Vitamin D conc (ng/mL)**	17.4 (±0.59)	17.5 (±0.10)	0.907
**SBP (mmHg)**	119.8 (±1.60)	117.3 (±0.17)	0.111
**DBP (mmHg)**	73.4 (±0.81)	76.4 (±0.12)	<0.001
**BMI (kg/m^2^)**	21.9 (±0.29)	23.7 (±0.03)	<0.001
**Total energy intake**	1704.4 (±73.3)	2056.5 (±9.12)	<0.001
**Calcium intake (mg)**	537.4 (±64.3)	514.1 (±3.14)	0.718
**Household income, No. (%) of participant**			<0.001
**Low**	82 (28.7)	6272 (14.3)	
**Low-middle**	53 (29.8)	8396 (26.1)	
**High-middle**	45 (21.0)	9039 (29.8)	
**High**	38 (20.5)	9194 (29.7)	
**Alcohol consumption, No. (%) of participant**			<0.001
**Never**	125 (53.4)	9523 (22.5)	
**≤1 times/month**	32 (15.3)	9742 (29.4)	
**2–4 times/month**	24 (16.0)	6986 (24.8)	
**≥2 times/week**	37 (15.3)	6650 (23.3)	
**Education (%), No. (%) of participant**			<0.001
**≤Elementary**	106 (37.3)	8529 (17.1)	
**Middle school**	35 (17.3)	3649 (9.6)	
**High school**	52 (28.7)	11,075 (39.7)	
**≥College**	25 (16.7)	9648 (33.6)	
**Smoking status, No. (%) of participant**			<0.001
**Never**	81 (37.5)	20,022 (53.4)	
**Former**	72 (36.1)	4377 (14.7)	
**Current**	65 (26.4)	8502 (31.9)	

Survey regression and survey (Rao–Scott) χ^2^ test were used for and continuous and categorical variables, respectively.

**Table 2:** General characteristics stratified by total serum 25-hydroxyvitamin D (25(OH)D) concentrations.

	Total 25(OH)D Concentrations (ng/mL)			
**Characteristic**	<12 (n = 5927)	12–19.99 (n = 16,620)	≥20 (n = 10,572)	*p*
**Sex, No. (%) of participant**				<0.0001
**Men**	1648 (38.7)	6452 (49.8)	5421 (61.2)	
**Women**	4279 (61.3)	10,168 (50.2)	5151 (38.8)	
**Age (years)**	40.97 (±0.31)	43.44 (±0.20)	48.77 (±0.28)	<0.0001
**Vitamin D conc. (ng/mL)**	9.91 (±0.03)	15.83 (±0.03)	25.22 (±0.09)	<0.0001
**SBP (mmHg)**	115.6 (±0.35)	116.8 (±0.20)	119.3 (±0.28)	<0.0001
**DBP (mmHg)**	75.3 (±0.24)	76.3 (±0.14)	77.3 (±0.20)	<0.0001
**Total energy intake**	1910.2 (±16.2)	2062.1 (±12.4)	2137.2 (±14.9)	<0.0001
**Calcium intake (mg)**	466.1 (±5.87)	515.2 (±4.07)	544.1 (±5.52)	<0.0001
**Household income, No. (%) of participant**				<0.0001
**Low**	1003 (14.1)	2838 (13.1)	2513 (16.9)	
**Low-middle**	1558 (27.1)	4215 (26.0)	2676 (25.7)	
**High-middle**	1656 (30.0)	4752 (30.6)	2676 (28.2)	
**High**	1710 (28.8)	4815 (30.4)	2707 (29.1)	
**Alcohol consumption, No. (%) of participant**				<0.0001
**Never**	1922 (25.6)	4633 (21.9)	3093 (22.2)	
**≤1 times/month**	2012 (34.1)	5040 (29.6)	2722 (25.6)	
**2–4 times/month**	1193 (23.9)	3702 (25.8)	2115 (23.4)	
**≥2 times/week**	800 (16.4)	3245 (22.8)	2642 (28.8)	
**Education (%), No. (%) of participant**				<0.0001
**≤Elementary**	1241 (13.2)	3819 (15.1)	3575 (23.5)	
**Middle school**	526 (7.3)	1717 (8.9)	1441 (12.4)	
**High school**	2168 (42.8)	5813 (40.4)	3146 (36.2)	
**≥College**	1992 (36.7)	5271 (35.6)	2410 (27.8)	
**Smoking status, No. (%) of participant**				<0.0001
**Never**	4062 (60.5)	10,382 (54.8)	5659 (46.1)	
**Former**	552 (10.6)	2247 (14.7)	1650 (17.7)	
**Current**	1313 (28.9)	3991 (30.5)	3263 (36.2)	
**Body mass index (%), No. (%) of participant**				<0.0001
**Underweight**	381 (7.0)	680 (4.3)	395 (3.7)	
**Normal weight**	2666 (45.6)	6629 (39.9)	4023 (37.6)	
**Overweight**	1233 (19.5)	3910 (22.9)	2702 (25.4)	
**Obesity**	1647 (28.0)	5401 (32.9)	3452 (33.3)	
**Frequency of walking**				<0.0001
**Never**	944 (13.7)	2454 (13.4)	1614 (14.8)	
**1–2 times/week**	1108 (19.2)	2908 (17.7)	1554 (15.6)	
**3–4 times/week**	1189 (19.1)	3467 (20.6)	1975 (18.6)	
**5–6 times/week**	981 (18.1)	2670 (17.1)	1505 (15.0)	
**Every day**	1705 (29.8)	5121 (31.2)	3924 (36.1)	

Survey (Rao–Scott) χ2 test was used for categorical variables.

**Table 3:** Adjusted odds ratios and 95% confidence intervals of gastric cancer stratified by total 25(OH)D concentrations.

Variables	No. Stomach Cancer/No. Participants	Model A	Model B
Total 25(OH)D (ng/mL)			
5-ng/mL increased	218/33,119	0.813 (0.698–0.947) **	0.839 (0.719–980) *
<12(deficient)	41/5927	1 (Reference)	1 (Reference)
12–19.99 (suboptimal)	96/16,620	0.669 (0.384–1.166)	0.681 (0.390–1.186)
≥20(sufficient)	81/10,527	0.520 (0.294–0.919) *	0.568 (0.322–1.002) ^§^
p-trend		0.030	0.065

** *p* < 0.001, * *p* < 0.05, ^§^ *p* < 0.1 compared with reference group (vitamin concentrations <12 nmol/L). Model A was adjusted for age, sex, body mass index. Model B: model A further adjusted for education, household income, smoking status, alcohol consumption and dietary factors (intake of total energy, calcium, vitamin A).

## Supplementary Materials

The following are available online at https://www.mdpi.com/2072-6643/12/7/2004/s1, Table S1: Adjusted odds ratios and 95% confidence intervals of gastric cancer stratified by total 25(OH)D concentrations (current status).
