# Supplementary material for: Vitamin D Status and Gastric Cancer: A Cross-Sectional Study in Koreans
Source: Nutrients. 2020 Jul 6;12(7):2004. doi: 10.3390/nu12072004 (PMC7400919; doi:10.3390/nu12072004)
Supplement: Supplementary file 1 [file nutrients-12-02004-s001.pdf]

Supplemental Table 1. Adjusted odds ratios and 95% confidence intervals of gastric cancer stratified by total 25(OH)D concentrations (current status)

Variables	No. stomach cancer/No. participants	Model A	Model B
Total 25(OH)D (ng/mL)			
5 ng/mL increased	72/33119	0.974 (0.799–1.188)	1.056 (0.856–1.304)
<12 (deficient)	7/5927	1 (Reference)	1 (Reference)
12–19.99 (suboptimal)	33/16620	1.001 (0.335–2.991)	0.979 (0.330–2.910)
≥20 (sufficient)	32/10527	1.041 (0.350–3.093)	1.231 (0.411–3.574)
p trend		0.928	0.701

**p<0.001, *p<0.05, [§]p<0.1 compared with reference group (vitamin concentrations <12 nmol/L). Model A was adjusted for age, sex, body mass index. Model B: model A further adjusted for education, household income, smoking status, alcohol consumption, and dietary factors (intake of total energy, calcium, vitamin A).
